# Study of some potential biomarkers in Egyptian hepatitis C virus patients in relation to liver disease progression and HCC

**DOI:** 10.1186/s12885-023-11420-1

**Published:** 2023-10-05

**Authors:** Kholoud Baraka, Rania R. Abozahra, Eman Badr, Sarah M. Abdelhamid

**Affiliations:** Microbiology and Immunology Department, Faculty of Pharmacy, Damanhour University, El Gomhoreya Street, Damanhour, El Behira Egypt

**Keywords:** Cytokines, miRNA, HCV, Cirrhosis, PCR

## Abstract

**Background:**

Egypt has the greatest prevalence of hepatitis C worldwide according to the WHO reports, accounting for 13% of the global HCV infections. HCV is a substantial precursor for fibrosis, cirrhosis, and hepatocellular carcinoma. This study aimed to investigate the potential relevance of some cytokines, miR-122 and miR-221 for the diagnosis of liver disease progression associated to HCV infection.

**Methods:**

One hundred and twenty blood samples were collected from patients with chronic liver disease, HCC, and healthy individuals. Total bilirubin, alanine aminotransferase, aspartate aminotransferase, platelet count, albumin, and creatinine were measured. Serum level of selected cytokines was conducted by ELISA. Serum miRNA expression was detected by RT-PCR.

**Results:**

IL-2R was higher among HCC patients and the mean concentration of both TNF-αRII and IL-6R was higher among cirrhotic patients. The expression of miRNA-122 showed a little fold decrease in all studied groups; the highest level was observed in HCC patients. The expression of miRNA-221 showed a significant fold increase in HCC and cirrhotic groups.

**Conclusions:**

This study revealed that there is no difference in liver disease progression in patients regarding sex and age. Routine liver function tests performed poorly in terms of early diagnosis of liver disease progression; however, serum total bilirubin gave somewhat useful guide for discrimination between fibrotic, cirrhotic and HCC cases. IL-2R showed a significant consistent increase in its level with disease progression. The miR-221 serum level showed significant fold increase with liver disease progression. Therefore, making miR-221 a potential non-invasive biomarker for liver disease progression in the diagnostic setting is recommended.

## Background

HCV is a hepatotropic RNA virus belonging to *Hepacivirus* genus, *Flaviviridae* family. It is an enveloped virus containing a 50 nm encased positive RNA strand. HCV is divided into six primary genotypes, each of which has a nucleotide sequence that differs by at least 30% from others. This genetic heterogeneity among the population serves as a significant selection mechanism for medication resistance and immune system evasion. In Egypt, HCV genotype 4 is the predominant genotype ( 92.5%) followed by genotype 1 (3.6%) [1, 2]. According to the WHO reports, Egypt has the greatest prevalence of hepatitis C in the world, accounting for 13% of the global population’s HCV infections. The primary source of its dissemination was a huge anti-bilharziasis injection therapy campaign that took place between 1960 and 1980. It has been found that the syringes used in this campaign were either improperly sterilized or were not sanitized at all. This could explain why the prevalence of hepatitis C is so high among persons over the age of 40 in some rural areas [3]. In its acute infection stage, HCV infection is largely silent, with few visible symptoms. Although roughly 30% of individuals may be able to clear the virus on their own [4], HCV is a substantial “precursor” for fibrosis, cirrhosis, and eventually hepatocellular carcinoma (HCC), in chronic instances [5, 6]. In Egypt, up to 85% of HCV infections have led to chronic hepatitis [4, 5]. Liver cirrhosis, liver failure, hepatic encephalopathy, and hepatocellular carcinoma, are the main leading causes of death [4, 6]. Despite the ministry of health’s efforts to discover HCV-infected persons and provide appropriate treatment, Egypt remains one of the top ten nations with the greatest HCV incidence internationally [9] representing a significant health and economic burden. Detection of asymptomatic people, providing them with care, and affording cheap therapies are all obstacles to attain HCV worldwide elimination targets [7].

According to the latest Global Cancer Statistics (GLOBOCAN 2020), hepatocellular carcinoma is the sixth most frequent cancer worldwide, with more than 900,000 estimated annual new cases (4.7%). Although its risk factors are well-known, it accounts for 8.3% of deaths of all cancers globally, being the third leading cause of worldwide cancer death [8]. It is estimated that, by 2025, more than one million individuals will have liver cancer annually [9]. Moreover, hepatocellular carcinoma accounts for 75–85% of the liver cancer [10]. It is the fourth prevalent cancer in Egypt [11]. The increased incidence of HCC could be attributed to improved screening programs and diagnostic tools [14], increased survival rate of cirrhotic patients, which increases the risk of developing HCC, and [3] increased incidence and complications of HCV, which is the most important risk factor in developing liver cancer, including HCC in Egypt [16].

Regarding the effectiveness of direct acting antiviral (DAA) medication in preventing HCC recurrence, many studies reported heterogenous results. In HCV-infected individuals treated with DAAs, several studies have reported an unexpectedly high prevalence of early HCC recurrence [17–19]. As a result, a precise assessment of HCC risk factors is critical for well-designed HCC preventive measures.

MicroRNAs (miRNAs) are a group of endogenous, noncoding, functional RNAs that are tiny (about 22 nucleotides in length) [20]. Some miRNAs are expressed universally, while others are tissue-specific [12, 13]. Disease state can be linked to changes in miRNA expression within a tissue type [23, 24]. Many studies have shown that certain forms of miRNA profiling can identify HCC among HCV-infected patients [14–16].

Cytokines are secreted or membrane-bound proteins that govern the development, differentiation, and activation of immune cells. They are one of the immune system components that contribute in the host response to invading infections. Infection, inflammation, and carcinogen-induced damage are all examples of cellular stressors that cause cytokines to be released. Several cytokines, especially those produced by CD4+ (cluster of differentiation 4) Th cells (T helper cells), are classified as Th1 or Th2 cytokines and they mostly consist of interleukins (ILs). Th1 cytokines (e.g., IL-1, IL-2, IL-12p35, IL-12p40, IL-15, and non-ILs, such as: Tumor Necrosis Factor (TNF) and interferons (IFN) are pro-inflammatory, whereas Th2 cytokines (e.g., IL-4, IL-8, IL-10, and IL-5) generate anti-inflammatory responses [17]. Many cell types in the liver are sensitive to the effects of cytokines. Receptors for IL-1, TNF-, and IL-6 are among the cytokine receptors found on hepatocytes. As a result, cytokines have been linked to liver development and regeneration, as well as the pathogenesis of liver disorders such as: cirrhosis, fibrosis, and cancer. Immunohistochemistry (IHC), quantitative real-time PCR (qRT-PCR), and enzyme linked immuno-sorbent assay (ELISA) can all be used to detect cytokines’ levels in both serum and plasma [17, 18].

## Materials and methods

### Patient criteria and sample collection

Sixty blood samples were collected from newly diagnosed HCV patients during the initiative of the president of the republic to eliminate HCV infection in 2018 from Damanhour Medical National Institute, thirty blood samples were collected from HCC patients at Damanhour Oncology Institute, and 30 blood samples were collected from healthy volunteers (with normal transaminases, normal hepatic ultrasound and negative for HBsAg, HBc-Ab and HCV RNA-PCR). Blood samples were divided into 4 groups: **Group I**; 30 patients with HCV related HCC diagnosed by Triphasic computed tomography (CT) scan with or without elevated Alpha-Fetoprotein (AFP), **Group II**; 30 chronic HCV patients with compensated cirrhosis (Child-Pugh class A, FIB-4 scores 3 or 4), **Group III**; 30 chronic HCV patients without cirrhosis (Child-Pugh class A, Fibrosis-4 (FIB-4) scores 1 or 2), and **Group IV**; 30 healthy volunteers as a control group. Exclusion criteria of patients included: HCV patients receiving antiviral therapy, Co-infected with other hepatitis viruses, or Co-infected with any other viral infection. Five ml of blood were collected from each patient, and sera were separated by centrifugation and stored at -80 ^o^C for further use. Laboratory reports of serum alanine aminotransferase (ALT), aspartate aminotransferase (AST), platelet count, serum albumin, serum creatinine and total bilirubin for all patients were collected.

### Cytokines assay using ELISA

Interleukin-2, IL-6, and TNF-α were assayed for all study groups by quantitative ELISA plate method using the following ELISA kits according to the manufacturer’s instructions: Human Interleukin-2 receptor (IL-2R) (Beijing, China), soluble Human Interleukin-6 receptor (IL-6R) (Beijing, China), Human soluble tumor necrosis factor alpha II receptor (sTNF-α RII) (Beijing, China). The cut-off values of the studied cytokines were calculated according to the following equation: cut-off value = mean + 2SD of the negative controls. ELISA kits used Sandwich-ELISA method. The ELISA microtiter plate provided in the kit had been pre-coated with an antibody specific to the targeted Interleukin. Standards and samples were added to the appropriate ELISA microtiter wells to combine to the specific antibody. Then, a Horseradish Peroxidase (HRP) - conjugated antibody specific for the targeted interleukin was added to each ELISA microtiter well and incubated. Free components were washed away. The 3, 3, 5, 5’- tetramethylbenzidine (TMB) substrate solution was added to each well. Only wells that contained the interleukin and HRP conjugated IL- antibody appeared blue in color and then turned yellow after the addition of the stop solution. The optical density (OD) was measured spectrophotometrically at a wavelength of 450 nm. The OD value was proportional to the concentration of targeted interleukin. The concentrations of interleukins in samples were calculated by comparing the OD of samples to the standard curves.

### MiRNAs extraction and reverse transcription

MiRNAs 122 and 221 were extracted from the sera of patients and control group using Qiagen ® miRNeasy Mini Kit (Qiagen, Valencia, CA, USA) according to the manufacturer’s instructions. The RNA purity was assessed by the RNA concentration by the NanoDrop Jenway (Nanodrop, UK, Cole-Parmer Ltd). Single-stranded cDNAs were generated using miRCURY LNA Reverse Transcription Kit (cat. no. 339,340) according to the manufacturer’s protocol.

### MiRNA quantification using real-time PCR

Complementary-DNA that had been prepared in a reverse-transcription reaction served as the template for quantitative real-time PCR analysis using miRCURY LNA miRNA PCR Panels, the miRCURY LNA SYBR® Green PCR Kit, and Real-Time PCR thermal cycler (Applied Biosystems; StepOne^™^ Real-Time PCR, Foster City, CA, USA). The housekeeping miR-16-5P (5’GTTCCACTCTAGCAGCACGTAAATATTGGCGTAGTGAAATATATATTAAACACCAATATTACTGTGCTGCTTTAGTGTGAC3’) [19] was used as the endogenous control. Relative expression of miRNA-122 and miRNA-221 were calculated using the comparative cycle threshold method. The 2^-ΔΔCt^ method was used to determine relative-quantitative levels of individual miRNAs [20].

### Statistical analysis

Correlations between sex, age, liver function tests, platelet count, serum levels of the selected cytokines and serum levels of the two miRNAs in the four studied groups were statistically determined by using the Chi-square test and the IBM SPSS software package version 25.0. Significance of results was adjusted at level of 0.05.

## Results

### Demographic and clinic-pathological characteristics of patients and control group

This study was carried out on 120 participants classified into 4 groups as mentioned in materials and methods section. Demographic and clinic-pathological characteristics of patients and control group were illustrated in Table 1. Sixty-nine males and 51 females aged from20 to 73 years contributed in this study. Platelet count and liver function tests were carried out for patients and control group. Regarding serum alanine aminotransferase (ALT), normal values were detected among the control group individuals; however, higher abnormal values were detected among the fibrotic and HCC patients, and the highest values were detected among the cirrhotic group. Regarding Serum aspartate aminotransferase (AST), normal values were detected among the control group individuals; however, higher abnormal values were detected among the fibrotic, followed by the HCC group, and the highest values were detected among the cirrhotic group. Regarding platelet count, normal values were detected among the control and fibrotic groups’ individuals; however, lower abnormal values were detected among the cirrhotic group, followed by the lowest values in the HCC group. Regarding serum albumin, normal values were detected among the control, fibrotic and cirrhotic groups’ individuals; however, lower abnormal values were detected among the HCC group. Regarding serum creatinine, normal values of serum creatinine were detected among all the studied groups. Regarding serum total bilirubin, normal values were detected among all the studied groups.

**Table 1:** Demographic and clinicopathological characteristics of patients and control

Variables		HCC* (Gp I) (n = 30)			Cirrhotic (Gp II) (n = 30)		Fibrotic (Gp III) (n = 30)	Control (Gp IV) (n = 30)	Test of Significance	*P* value
		No. (%)			No. (%)		No. (%)	No. (%)		
Sex	Male	22 (73.33%)			15 (50%)		13 (43.33%)	19 (63.3%)	Chi-Square= 6.65	0.084
	Female	8 (26.67%)			15 (50%)		17 (56.67%)	11 (36.7%)		
Age	Min-Max	45–73			43–71		20–71	50–71	F = 0.146	0.932
	Mean ± SD	61.3 ± 7.2			61.9 ± 5.9		60.9 ± 11.5	62.1 ± 3.7		
	Median	60			61.5		64.5	61		
Serum ALT (U/L)	Min. - Max.	16–187			25.0- 174.0		20.0–242.5	14–40	F = 11.412*	0.000
	Mean ± SD.	63.13 ± 42.22			73.29 ± 39.3		54.45 ± 28.7	26.67 ± 7.82		
	Median	40			57.2		47.0	26		
	Sig Best Groups	P1 = 0.690, P2 = 0.783, P3 = 0.001, P4 = 0.174, P5 = 0.00, P6 = 0.014								
Serum AST (U/L)	Min. - Max.	7- 178		45.4–146.3		20.6–137.1		14–36	F = 15.393*	0.000
	Mean ± SD.	65.9 ± 50.58		72.1 ± 29.25		40.87 ± 14.87		25.53 ± 7.64		
	Median	68		69.05		40.45		28		
	Sig Best Groups	P1 = 0.889, P2 = 0.020, P3 = 0.000, P4 = 0.002, P5 = 0.000, P6 = 0.286								
Platelet count (Χ10^3^/μl)	Min. - Max.	35–165		51.0–193.0		101–429		180–410	F = 84.601*	0.000
	Mean ± SD.	110.40 ± 38.4		122.97 ± 29.8		211.6 ± 62.6		297.2 ± 66.93		
	Median	114		122		210		292		
	Sig Best Groups	P1 = 0.830, P2 = 0.000, P3 = 0.000, P4 = 0.000, P5 = 0.000, P6 = 0.000								
Albumin (g/dl)	Min. - Max.	2.2- 4		2.9–4.65		3.86–5.13		3.9–5.2	F = 103.080*	0.000
	Mean ± SD.	2.96 ± 0.52		4.097 ± 0.46		4.7 ± 0.3		4.67 ± 0.45		
	Median	2.85		4.265		4.675		4.9		
	Sig Best Groups	P1 = 0.000, P2 = 0.000, P3 = 0.000, P4 = 0.000, P5 = 0.000, P6 = 0.992								
Creatinine (mg/dl)	Min. - Max.	0.6–1.3		0.7–1.32		0.74–1.32		0.6–1.1	F = 4.127*	0.008
	Mean ± SD.	0.92 ± 0.26		0.87 ± 0.15		0.998 ± 0.17		0.83 ± 0.19		
	Median	0.9		0.86		0.995		0.75		
	Sig Best Groups	P1 = 0.870, P2 = 0.474, P3 = 0.362, P4 = 0.122, P5 = 0.823, P6 = 0.012								
Total bilirubin (mg/dl)	Min. - Max.	0.5–1.8	0.5–2.1			0.25–1.49		0.4–1.1	F = 22.572*	0.000
	Mean ± SD.	1.24 ± 0.25	0.9 ± 0.37			0.677 ± 0.277		0.76 ± 0.23		
	Median	1.3	0.8			0.62		0.8		
	Sig Best Groups	P1 = 0.000, P2 = 0.000, P3 = 0.000, P4 = 0.030, P5 = 0.303, P6 = 0.741								

- Sig Best Groups was done by using Scheffe for Multiple Comparisons

*P*1: p value for comparing the HCC group and Cirrhotic group

*P*2: p value for comparing the HCC group and Fibrotic group

*P*3: p value for comparing the HCC group and Control group

*P*4: p value for comparing the Cirrhotic group and Fibrotic group

*P*5: p value for comparing the Cirrhotic group and Control group

*P*6: p value for comparing the Fibrotic group and Control group

*: Statistically Significant at *P* ≤ 0.01

*HCC: hepatocellular carcinoma

### Serum levels of cytokines

Interleukin-2, IL-6, and TNF-α were assayed for all study groups by quantitative ELISA plate method. Concentration of IL-2R was higher among HCC patients than in other groups (308.1 ± 193.1 ng/ml). There was a consistent increase in the IL-2R level with the disease progression from control to HCC. On the other hand, the mean concentration of TNF-αRII was higher (89.7 ± 56.8 ng/ml) among cirrhotic patients than in other groups. Moreover, IL-6R was significantly higher among cirrhotic patients than fibrotic and HCC patients (Table 2).

**Table 2:** Serum levels of IL-2, IL-6, and TNF-α among the studied groups (mean ± SD)

Cytokines		Fibrotic (n = 30)	Cirrhotic (n = 30)	HCC (n = 30)
IL-2R	Cutoff value = 3.03 ng/ml No. of positive cases	3.4 ± 1.7 15 (50%)	4.1 ± 2.1 19 (63%)	308.1 ± 193.1 30 (100%)
IL-6R	Cutoff value < 0.9 ng/ml No. of positive cases	3.35 ± 2.8 17 (56.7%)	4.4 ± 3.6 13 (43.3%)	< 3 ± 0 30 (100%)
TNF-αRII	Cutoff value < 13.5 ng/ml No. of positive cases	51 ± 22.3 4 (13.3%)	89.7 ± 56.8 9 (30%)	26.4 ± 12.9 9 (30%)

### MiRNA-122 serum levels

The analysis of median fold change in the expression level of miRNA-122 in patients’ sera in comparison to the control group showed a little fold decrease in expression in all studied groups. The highest level was observed in HCC patients followed by cirrhotic then fibrotic patients with median fold change 0.8, 0.4 and 0.2 respectively. There was no significant statistical difference between the three groups regarding MiRNA-122 compared to the control group (*P* = 0.372) (Table 3).

### MiRNA-221 serum levels

The analysis of fold change in the expression level of miRNA-221 in patients’sera in comparison to the control group showed a significant fold increase in HCC and cirrhotic groups. In this study, a significant fold increase was found in the serum mi-221 levels of HCC patients in comparison to cirrhotic group and to fibrotic group. There was statistically significant difference between the three groups regarding MiRNA-221 (*P =* 0.000). A statistically significant difference between the HCC group and the cirrhotic (*P* = 0.000) was found. Moreover, there was a significant difference in MiRNA-221 level between the HCC group and fibrotic group (*P* 0.000). Whereas, no statistically significant difference was detected between patients with cirrhosis and fibrosis (*P* = 0.745) (Table 3).

**Table 3:** miRNA-122 and miRNA-221 serum levels in the different studied groups

Variables	Group			F(test)	*P* value
	HCC (n = 30)	Cirrhotic (n = 30)	Fibrotic (n = 30)		
Median of fold change compared to control					
MiRNA-122	0.817	0.4097	0.23	1.0	0.372
MiRNA-221	31.2807	5.7645	1.17915	15.442*	0.000
	Sig Best Groups				
	P1 = 0.000, P2 = 0.000, P3 = 0.745				

- Sig Best Groups was done by using Scheffe for Multiple Comparisons

*P*1: p value for comparing between HCC group and Cirrhotic group

*P*2: p value for comparing between HCC group and Fibrotic group

*P*3: p value for comparing between Cirrhotic group and Fibrotic group

*: Statistically Significant at p ≤ 0.01

## Discussion

Egypt was considered the country with the highest HCV prevalence in the world in 2008 according to the Egyptian Demographic Health Survey (EDHS) which was conducted in that year [21, 22]. Patients with chronic hepatitis C (CHC) are at a significant risk of developing life-threatening consequences such as: cirrhosis and hepatocellular cancer (HCC) [23, 24]. In HCV-infected individuals treated with DAAs, multiple studies have found an unexpectedly high prevalence of early HCC recurrence [25–27]. In 2018, a program had grown into a national strategy to eradicate HCV as a public health threat. This new policy was developed in line with the first Global Health Sector Strategy on Viral Hepatitis 2016–2021, which was unanimously endorsed by the 194 WHO member states, including Egypt. By 2030, WHO signatories pledged to eradicate viral hepatitis as a public health issue [28]. Despite the fact that Egypt had treated over 2 million patients since 2014, HCV infection was still a big concern when that program began in 2018, with 4.6% of the previously untreated adult population seropositive. Egypt remained one of the ten countries with the greatest HCV burden in the world, posing a significant health and economic burden [9].

It’s crucial to remember, though, that widespread DAA treatment will not alleviate all of Egypt’s HCV epidemic’s difficulties. Decompensated cirrhosis and hepatocellular carcinoma consequences are a significant burden on Egyptian society, and they must be managed with sufficient health-care resource allocation [24]. Hepatocellular carcinoma is frequently asymptomatic and invasive in its early stages. Because the majority of HCC patients have non-operable disease, early detection is crucial for a favorable prognosis. Early identification of HCC allows for curative treatments such as: liver transplantation, resection, or local ablative therapies which are the most effective ways to extend survival. As a result, ongoing research is being conducted around the world to identify and test an early, sensitive, and specific marker for HCC diagnosis [29].

Clinical information, hepatic ultrasonography, and serum alpha-fetoprotein (AFP) monitoring at 6–12 month intervals are used to diagnose HCC, although neither of these methods is adequate for detecting extremely tiny (2 cm) HCC tumors. Alpha-fetoprotein has a limited sensitivity, especially when it comes to early-stage HCC detection. Furthermore, many people with non-malignant chronic liver disease have elevated AFP levels in their blood. As a result, innovative biomarkers with great efficacy for early identification and therapeutic monitoring of HCC are urgently needed [30].

Many cell types in the liver are sensitive to the effects of cytokines. IL-1, TNF-, and IL-6 are among the cytokine receptors found on hepatocytes. Non-parenchymal cells, such as: resident liver macrophages (Kupffer cells), not only produce a variety of cytokines, but their functions are also influenced by the cytokine environment. As a result, cytokines have been linked to liver development and regeneration, as well as the pathogenesis of liver illnesses such as cirrhosis, fibrosis, and cancer. Immunohistochemistry (IHC), quantitative real-time PCR (qRT-PCR), and ELISA can all be used to detect cytokines in the serum and plasma [17].

In the present study, IL-2R was above normal in 100% of HCC and in 63% of cirrhotic cases in our study. The concentration of IL-2R was higher in HCC patients than in other groups and that was fully agreed with what was reported by Zhong et al. [31]. Moreover, Iwane et al. [32] and Chengwen et al. [33] reported significant correlation between high level IL-2R and liver carcinoma. In individuals with chronic HCV infection, serum levels of sIL-2R correlate with the histological degree of liver damage and could be utilized as a marker in patients at high risk, this theory was supported by Izzo et al. [34, 35]. Patients with high levels of soluble IL-2R, had the highest risk of developing HCC. The sIL-2R may act as a sink for IL-2, resulting in lower levels of this cytokine. This would favor tolerance induction over immunity maintenance, implying that sIL-2R plays a key role in the negative feedback mechanism that restores the balance between immunity and tolerance [36].

In the present study, IL-6R level was significantly higher in HCC than the control group. Its level was above normal in 100% of HCC cases and there was no significant difference in its level between cirrhotic and fibrotic cases. Similarly, Zhang et al. [37] reported that high expression level of IL-6R promoted HCC recurrence. In order to promote HCC, IL-6 binds to IL-6R on the target-cell surface and activates the Janus kinase/signal transducers and activators of transcription (STAT) pathway [37]. In the liver, IL-6 is known to have a pleiotropic effect. Its traditional functions include infection defense and hepatocyte homeostasis, as well as acting as a mitogen; nevertheless, inappropriate or persistent IL-6 signaling can cause inflammatory disorders, metabolic problems, and even liver cancer [38]. Binding of that cytokine to its receptor (IL-6R) produces a set of complicated signals which were proven to be tumor enhancement. Furthermore, it was shown that IL-6 exposure could lead to an increase in the expression of IL-6R, potentially leading in a feedback loop that amplifies the IL-6-induced impact [39].

TNF, a pro-inflammatory cytokine, is involved in the pathophysiology of liver disease. Over the last years, specific antagonists for this cytokine had been discovered. TNF soluble receptors RI and RII, which are formed from the cell surface, are naturally occurring molecules that inhibit tumor necrosis factor’s biological effects [40]. The extracellular domains of the receptors are cleaved, resulting in soluble versions of sTNF-αRs. Although both forms of sTNF-αRs (RI and RII) are found in normal serum, sTNF-αRII is more plentiful and has a higher affinity for TNF-α than sTNF-αRI, implying that sTNF-αRII is more essential in reducing TNF-α activity [41]. Kakumu et al. [41] reported that TNF-αRII was higher in cirrhotic and HCC patients than in control group and that was similar to our results. In addition, Chia et al. [42] reported that several pro-inflammatory cytokines, including TNF-αRII, levels had been found higher in patients with HCC as compared to healthy controls. On the other hand, Zekri et al. [40] reported that, HCC patients had greater levels of IL-2R, IL-6R and IL-10 than other groups. In contrast, TNF-RII mean concentration in this study was higher in cirrhotic patients than in other groups. The increase in TNF-RII concentration in our patients may reveal that HCV-related liver disease involves immunological mechanisms, which including TNF system activation, and may reflect the degree of inflammation and progression of HCC.

Non-coding RNAs account for a large component of the human genome. They’ve long been known to serve crucial roles in maintaining cellular homeostasis and functionality. Some non-coding RNAs have cell- and tissue-specific expression patterns and are deregulated specifically in pathological situations (e.g. cancer). Non-coding RNAs have been investigated as potential biomarkers in the context of many diseases for several years, with a focus on microRNAs (miRNAs) and long non-coding RNAs (lncRNAs). MiRNAs, either as transcriptional or post-transcriptional regulators, have been shown to play a significant role in controlling gene expression [43]. Humans create between 2,000 and 3,000 miRNAs. Many miRNAs are expressed throughout all tissues, while others are tissue-specific [13].They control post-transcriptional gene expression by inhibiting translation and/or destabilizing mRNA. Because miRNAs are involved in so many developmental processes, it’s very likely that changes in their expression are linked to abnormal circumstances like poor growth or illnesses. As a result, miRNA expression can be employed as a biomarker in pathophysiological circumstances [44].

The MiR-122 is prevalent in the liver (tissue-specific) and is required for the stability and proliferation of HCV RNA. There are two spaces named S1 and S2 in the highly conserved 5′ UTR of the HCV genome that miR-122 can bind to. This culminates in the creation of an oligomeric miR-122-HCV complex, which protects the HCV genome from breakage and the host’s innate immune system [45]. This binding site is present in all kinds and subtypes of the HCV genome. As a result, miR-122 could be employed as a target in antiviral therapy [45]. Miravirsen is a 15-antisense oligonucleotide that has been engineered to be complementary to and highly specific for the mature miR-122 5′ region. Miravirsen can thereby isolate and inhibit miR-122. There was a drop in HCV RNA levels, but no evidence of viral resistance [46].

In the present study, results showed a significant fold decrease in expression level of miR-122 in HCC, cirrhotic and fibrotic groups in comparison to normal control group. Similarly, Zhou et al. [47] and Tan et al. [48] reported that MiR-122 was under-expressed in HCC serum or plasma samples as compared to normal controls in two genome-wide profiling studies. In contrast, Xu et al. [49] and Qi et al. [50] reported that circulating miR-122 was up-regulated in HCC compared to the control group. After combining miRNA expression in chronic hepatitis B (CHB) plasma samples, Qi et al. reported that MiR-122 is a good candidate biomarker for early liver pathology, but not notably for HCC [50]. In another study investigating three independent groups comprising a total 116 HCC patients and 79 NH individuals, it was reported that 12 miRNAs are differentially expressed between HCC and NH individuals in all three groups. Five up-regulated miRNAs (miR-122-5p, miR-125b-5p, miR-885-5p, miR-100-5p and miR-148a-3p) in CHB, cirrhosis and HCC patients are potential biomarkers for CHB infection [51].

The MiR-221 is an oncogenic microRNA. It increases cell growth and inhibit apoptosis. Additionally, a prior study showed that miR-221 enhanced tumor development, growth and metastasis in liver cancer, which had a negative impact on treatment outcomes [52]. In the present study, after the analysis of fold change in the expression level of miRNA-221 in patients’ sera in comparison to the control group, we found a significant fold increasing in the serum mi-221 levels of HCC patients in comparison to cirrhotic group and fibrotic group. Similarly, Ding et al. reported elevated levels of miR-221 in the serum of HCV-infected patients [53]. However, the mechanism of up-regulation of miR-221 remained unclear. The researchers revealed a correlation between elevated levels of miR-221, ALT, and AST. Indeed, it had been proven in other investigations that NF-kB was shown to influence the expression of miR-221. These results were also confirmed by Xu et al. [54].

On the other hand, Sohn et al. [55] reported that individuals with HCC and persistent HBV infection had high levels of miR-221 in serum exosomes and as a circulating miRNA mentioning that authors did not link tissue expression with blood levels of miR-221. Li et al. discovered significantly elevated levels of miR-221 in serum samples among HCC patients, and they demonstrated a relationship between the miR-221 concentration and the severity of the disease. Liver cirrhosis, tumor size, and stage were more advanced in patients with high serum levels of miR-221. Furthermore, the survival rate was noticeably lower than in patients with low serum levels of miR-221. However, authors suggested that using blood levels of both miR-221 and AFP together offered a more accurate diagnostic prediction than using either of these indicators alone [56]. Moreover, in a scientific report done by Nagy et al. [57], they revealed that, Hsa-miR-221 was among the top miRNAs showing substantial expression alterations when comparing HCC to normal liver tissue across three separate datasets. Its expression was higher in HCC tissues than in non-tumor tissues and that was compatible with our results.

## Conclusion

In Egypt, up to 85% of HCV infections last for a lifetime, resulting in chronic hepatitis. Liver cirrhosis, liver failure, hepatic encephalopathy, and hepatocellular carcinoma, are leading causes of death. HCC represents a significant health and economic burden in our country. HCC recurrence is more frequent so a precise assessment of HCC risk factors is critical for well-designed HCC prevention measures. Our study revealed that there is no difference in liver disease propagation and progression in patients regarding sex and age. Routine liver function tests such as: ALT, AST, serum creatinine, and albumin performed poorly in the surveillance mode and early detection of liver disease progression; however, serum total bilirubin gave somewhat useful guide for discrimination between fibrotic, cirrhotic and HCC patients. Even AFP was rejected either for the surveillance or the diagnosis of HCC according to the Practice Guidelines of the American Association for the Study of Liver Diseases (AASLD) (July 2010). In the current study, only IL-2R showed a significant consistent increase in its level with disease progression from control to HCC. Our study evaluated the signature of two circulating miRNAs (miR-122 and miR-221), and we found that level of miR-122 decreased -insignificantly- in the serum of HCC, cirrhotic and fibrotic groups compared to control. On the other hand, the serum level of miR-221 showed significant fold increase with liver disease progression from control to HCC. Thus, making miR-221 a potential non-invasive biomarker for liver disease progression in the diagnostic setting is recommended.

## Data Availability

All data generated or analyzed during this study are included in this published article.

## List of abbreviations

AFP: Alpha-Fetoprotein
ALT: Aminotransferase
AST: Aspartate aminotransferase
CD4: Cluster of differentiation 4
cDNA: Complementary deoxy-ribonucleic acid
CHC: Chronic hepatitis C
CT: Computed tomography
DAA: Direct acting antiviral
EDHS: Egyptian Demographic Health Survey
ELISA: Enzyme linked immuno-sorbent assay
IFN: Interferons
IHC: Immunohistochemistry
IL: Interlukin
HCC: Hepatocellular carcinoma
HCV: Hepatitis C virus
HRP: Horseradish Peroxidase
miR: Micro RNA
OD: Optical density
PCR: Polymerase chain reaction
RNA: Ribonucleic acid
RT: Reverse transcriptase
Th cells: T helper cells
TMB: Tetramethylbenzidine
TNF: Tumor Necrosis Factor
WHO: World health organization
